# Piceatannol-Loaded Emulsomes Exhibit Enhanced Cytostatic and Apoptotic Activities in Colon Cancer Cells

**DOI:** 10.3390/antiox9050419

**Published:** 2020-05-13

**Authors:** Nabil A. Alhakamy, Shaimaa M. Badr-Eldin, Osama A. A. Ahmed, Hani Z. Asfour, Hibah M. Aldawsari, Mardi M. Algandaby, Basma G. Eid, Ashraf B. Abdel-Naim, Zuhier A. Awan, Adel F. Alghaith, Ahmed L. Alaofi, Amir I. Mohamed, Solomon Z. Okbazghi, Mohammed W. Al-Rabia, Usama A. Fahmy

**Affiliations:** 1 Department of Pharmaceutics, Faculty of Pharmacy, King Abdulaziz University, Jeddah 21589, Saudi Arabia; nalhakamy@kau.edu.sa (N.A.A.); smbali@kau.edu.sa (S.M.B.-E.); oaahmed@kau.edu.sa (O.A.A.A.); haldosari@kau.edu.sa (H.M.A.); 2 Advanced Drug Delivery Research Group, Faculty of Pharmacy, King Abdulaziz University, Jeddah 21589, Saudi Arabia; 3 Center of Excellence for Drug Research and Pharmaceutical Industries, King Abdulaziz University, Jeddah 21589, Saudi Arabia; 4 King Fahd Medical Research Center, King Abdulaziz University, Jeddah 21589, Saudi Arabia; 5 Department of Pharmaceutics and Industrial Pharmacy, Faculty of Pharmacy, Cairo University, Cairo 11562, Egypt; 6 Department of Medical microbiology and Parasitology, Faculty of Medicine, King Abdulaziz University. Jeddah 21589, Saudi Arabia; hasfour@kau.edu.sa (H.Z.A.); mwalrabia@kau.edu.sa (M.W.A.-R.); 7 Department of Biological Sciences, Faculty of Science, King Abdulaziz University, Jeddah 21589, Saudi Arabia; malgandaby@kau.edu.sa; 8 Department of Pharmacology and Toxicology, Faculty of Pharmacy, King Abdulaziz University, Jeddah 21589, Saudi Arabia; beid@kau.edu.sa (B.G.E.); aaabdulalrahman1@kau.edu.sa (A.B.A.-N.); 9 Department of Clinical Biochemistry, Faculty of Medicine, King Abdulaziz University, Jeddah 21589, Saudi Arabia; zawan@kau.edu.sa; 10 Department of Pharmaceutics, College of Pharmacy, King Saud University, Riyadh 12372, Saudi Arabia; afalghaith@ksu.edu.sa (A.F.A.); ahmedofi@ksu.edu.sa (A.L.A.); 11 Department of Pharmaceutics and Industrial Pharmacy, Military Medical Academy, Cairo 11757, Egypt; alaa081866@miuegypt.edu.eg; 12 Global Analytical and Pharmaceutical Development, Alexion Pharmaceuticals, New Haven, CT 06510, USA; solomon.z.okbazghi@gmail.com

**Keywords:** piceatannol, emulsomes, HCT 116 cells, apoptosis, mitochondrial membrane potential, reactive oxygen species

## Abstract

Piceatannol (PIC), a naturally occurring polyphenolic stilbene, has pleiotropic pharmacological activities. It has reported cytotoxic activities against different cancer cells. In the present study, PIC emulsomes (PIC-E) were formulated and assessed for cytotoxic activity. A Box–Behnken design was employed to investigate the influence of formulation factors on particle size and drug entrapment. After optimization, the formulation had a spherical shape with a particle size of 125.45 ± 1.62 nm and entrapment efficiency of 93.14% ± 2.15%. Assessment of cytotoxic activities indicated that the optimized PIC-E formula exhibited significantly lower IC_50_ against HCT 116 cells. Analysis of the cell cycle revealed the accumulation of cells in the G2-M phase as well as increased cell fraction in the sub-G1 phase, an indication of apoptotic-enhancing activity. Staining of cells with Annexin V indicated increased early and late apoptosis. Further, the cellular contents of caspase - 3 and *Bax/Bcl-2* mRNA expression were significantly elevated by PIC-E. In addition, the mitochondrial membrane potential (MMP) was disturbed and reactive oxygen species (ROS) production was increased. In conclusion, PIC-E exhibited superior cell death-inducing activities against HCT 116 cells as compared to pure PIC. This is mediated, at least partly, by enhanced pro-apoptotic activity, disruption of MMP, and stimulation of ROS generation.

## 1. Introduction

Colorectal cancer (CRC) creates a high disease burden globally, and it has been projected to further increase by 60% by the year 2030 [1]. Surgery and/or chemotherapy are the standard treatments for this condition. Among many therapies presently available for colon cancer, herbal medicines are considered promising. In particular, natural products are gaining much attention as colon cancer chemotherapeutics [2]. A wide variety of chemical classes of plant active constituents have been reported for their potential role in colon cancer treatment. These include alkaloids and polyphenols [3]. Piceatannol (PIC) is a phenolic compound belonging to the stilbenoids class. It is a metabolite of resveratrol. Piceatannol has a plethora of pharmacological activities, including antitumor activity which has been found to be superior to resveratrol [4]. Piceatannol cytotoxicity has been reported against melanoma, leukemia, lymphoma, prostate, and colon cancer cells [5]. In addition, PIC synergizes the anti-cancer activities of cancer chemotherapeutics, thus overcoming drug resistance [6]. Gene modulation, inhibition of apoptosis protein, and mitochondrial fission are thought to underlie this activity. This further supports the claim that stilbenes act as multidrug resistance modulators and apoptosis inducers [7]. The anti-cancer action of PIC in colorectal cancer is promising. Promotion of apoptosis via upregulation of mRNA expression is provided as an explanation for this observation [8]. Nanotechnology has taken a prominent position in improving cancer chemotherapeutic agents. Interestingly, albumin nanoparticles of PIC have been reported effective in a murine model of colon cancer [9]. While all types of nanostructures are reported for use in the delivery of anti-cancer agents, lipid-based systems have some specific advantages. Their ability to cross biological barriers more efficiently than the polymeric nanostructures, owing to the lipophilic nature of the components, is one among them [10]. Lipid-based vesicular systems have been widely investigated in the pharmaceutical field as carriers to enhance the bioavailability of the drugs and/or promote their selective targeting to certain organ or tumor [11]. Liposomes are considered the first generation of such systems. Modified vesicular systems, structurally related to liposomes, have recently emerged to further improve the stability and delivery characteristics of such systems. These modified generations include niosomes, transfersomes, phytosomes, ethosomes, and emulsomes. Among these generations, emulsomes have the unique feature of combining the advantages of both liposomes and emulsions [12]. Also, emulsomes have some similarities to solid lipid nanoparticles and lipospheres. The combination of such features enables emulsomes to provide better solubility and bioavailability of wide variety drugs. Thus, they could be considered as a promising strategy for the formulation of PIC, which has both low solubility and poor oral bioavailability [9]. Furthermore, emulsomes have been increasingly investigated for their potential use in the delivery of chemotherapeutic agents in cancer therapy. Methotrexate-loaded emulsomes have been explored for lymph-targeted delivery [13]. Emulsomes have also been studied for enhancing solubility and tumor delivery of curcumin [14]. Piperine/curcumin dual emulsomes have also been explored as a combinational treatment strategy to enhance the cytotoxic effect in colorectal cancer. Modifications in the structure of emulsomes have also been studied for better tumor-targeted delivery. One example is attaching S-layer fusion proteins [12]. Modification by PEGylation and subsequent conjugation for better targeting is also a possible approach to achieve such an aim [15]. Thus, the present study aimed at developing and evaluating the cytotoxic potential of PIC-loaded emulsomes (PIC-E) in the colorectal cancer cells HCT 116.

## 2. Materials and Methods 

### 2.1. Materials

Piceatannol, cholesterol, and tristearin were purchased from Sigma–Aldrich Inc. (St. Louis, MI, USA). Lipoid^®^ S 100 (hydrogenated soybean lecithin, containing at least 90% phosphatidylcholine) was received as a gift from Lipoid GmbH (Frigenstr, Ludwigshafen, Germany). The HCT 116, HCT 29, and EA.hy926 cells were obtained from NCCS, Pune, India. All the other chemicals and reagents were of analytical grade.

### 2.2. Experimental Design

A Box–Behnken design was employed for the formulation of PIC-E. Two formulation factors and one process parameter were investigated as independent variables, namely, PIC concentration (%w/w, X1), Lipoid^®^ S 100 (%w/w, X2), and pH of hydration medium (X3). Particle size (nm, Y1) and entrapment efficiency (%, Y2) were studied as responses. The coded levels of each factor nominated as (−1, 0, +1) and their corresponding actual values are listed in Table 1. Design-Expert software (Version 12; Stat-Ease Inc., Minneapolis, MN, USA) was used to generate 17 experimental runs. The levels of variables for the experimental runs and their measured responses are presented in Table 2. Statistical analysis was performed to select the best fitting model for each response parameter based on the predicted and adjusted *R*^2^. Analysis of variance (ANOVA) was used to statistically analyze the responses, and statistical significance was at *p* ≤ 0.05. To explore the interactions among the studied variables, three dimensional plots were produced. 

#### 2.2.1. Preparation of PIC-E

To prepare PIC-E, a modified thin-film hydration technique was employed [13]. The PIC-E were prepared by dissolving specified quantities of PIC, Lipoid^®^ S 100, cholesterol (4% w/w), and tristearin (2% w/w) in 20 mL of a chloroform/methanol mixture (2:1, v/v) in a round-bottom flask. A rotavapor at 40 °C was used for the evaporation of the solvent under reduced pressure. To ensure that the organic solvent was removed, the deposited films were kept in a vacuum oven overnight for drying. Hydration of the residual traces was carried out with 10 mL phosphate buffer and gentle agitation at room temperature for 60 min. The PIC concentration, lipoid concentration, and pH of the hydrating buffer were specified for each run according to the experimental design. To produce nano-sized emulsomes, the resulting dispersion was ultra-sonicated at amplitude 40%, 750 W, 20 kHz (Sonics & Materials Inc., CT, USA) for 2 min in two cycles with five minutes between each cycle. The prepared emulsomes were kept at 4 °C until further investigations.

#### 2.2.2. Measurement of Particle Size

For determining the particle size of the prepared PIC-E, a dynamic light scattering technique was carried out using a particle size analyzer (Zetasizer Nano ZS90, Malvern, United Kingdom). 

#### 2.2.3. Drug Entrapment Determination 

The efficiency of PIC entrapment in the emulsomes was determined by analyzing the amount of drug content of emulsomes in comparison to the total amount added. For this purpose, a pre-weighed quantity of the emulsomes was subjected to disruption using n-propanol (50% v/v) in PBS (pH 7.4), and the amount of drug released drug was measured by HPLC [16]. The following formula was used to calculate the entrapment efficiency [13].

Entrapment efficiency (%) = (Determined PIC content)/ (Total PIC added) × 100.

#### 2.2.4. Optimization of PIC-E

The investigated formulation and process parameters were optimized using a numerical method following the desirability approach. The goal of the optimization process was minimizing vesicle size and maximizing drug entrapment.

### 2.3. Characterization of Optimized Formulation

#### 2.3.1. Transmission Electron Microscopy

The sample for transmission electron microscopy (TEM) was placed on a copper grid and stained using phosphotungstic acid. After removing the excess stain, the stained sample was dried and studied using the transmission electron microscope, JEOL-JEM-1011 (JEOL-Tokyo, Japan). 

#### 2.3.2. Drug Release (In Vitro)

The in vitro release of PIC from the emulsomes was studied following a reported method [9]. Phosphate-buffered saline (PBS, 0.1 M) at pH 7.4 containing Tween 80 (0.1%) was used in performing the release study. An accurately pre-weighed quantity of PIC-E containing 2 mg was introduced into a previously activated dialysis bag (MWCO=12,000 Da). The sample contained in the dialysis bag was maintained at 37 °C in a shaker water bath. The samples were removed at time points of 0.5, 1, 2, 4, 6, 8, 10, 12, 18, and 24 h and analyzed for PIC content by the same HPLC method which was mentioned previously in Section 2.2.3. The experiment was carried out in triplicate.

#### 2.3.3. Determination of IC_50_ Values

The procedure involved preparing HCT 116 and HCT 29 cells in 96 well plates at a cell density of 5 × 10^3^ per well cultured in McCoy’s 5A medium. While EA.hy926 was cultured in Dulbecco’s modified Eagle’s medium. Then, cells were incubated with different concentrations of plain-E, PIC or PIC-E using a range of concentrations with reference to PIC at logarithmic intervals for 48 h at 37 °C in a CO_2_ incubator. A commercially available MTT assay kit was used in determination of IC_50_ values based on the manufacturer’s instructions (ABCAM, Cambridge, UK). 

#### 2.3.4. Cell Cycle Analysis

A flow cytometer (FACSCalibur, BD Bioscience) was utilized in the determination of the cell cycle DNA distribution as previously described [17,18,19]. Briefly, six-well cell culture plates were seeded with approximately 5×10^3^ HCT 116 cells/well. Then, 0.1 µM PIC-E was added to the cells and equivalent concentrations of plain-E and plain-E for 24 h day. For cell cycle analysis, a CycleTEST™ PLUS DNA Reagent Kit (Becton Dickinson Immunocytometry Systems, San Jose, CA, USA) was used. The DI (DNA index) of the tested preparations was determined in reference to cells with a predetermined content of DNA. Staining was carried out using propidium iodide. Finally, CELLQUEST software (Becton Dickinson Immunocytometry Systems, San Jose, CA, USA) was used to study the distribution of the cell cycle.

#### 2.3.5. Annexin V Assay

The dual staining technique was performed to assess apoptosis as previously published [17]. The HCT 116 cells were incubated with plain-E, PIC-raw, and PIC -E with reference to 0.1 µM PIC in a six-well plate with a cell density of 5 × 10^3^ cells/well. A control sample with untreated cells was also included in the study. Staining was carried out using a commercially available kit (BD Bioscience, CA, USA). After incubation for 24 h, the cells were collected by centrifugation. The cells were then re-suspended in 500 μL of 1× binding buffer. Then, 5 μL each of annexin V-FITC and propidium iodide (PI) (BD Bioscience) were added and incubated at room temperature for 5 min in the dark. Flow cytometry (FACS Calibur, BD Bioscience) was employed for the analysis. MultiCycle software (Phoenix Flow Systems, San Diego, CA, USA) was used to study the results.

#### 2.3.6. Mitochondrial Membrane Potential

An assay kit (ABCAM, Cambridge, UK) was employed for the determination of mitochondrial membrane potential (MMP). This method exploited tetramethylrhodamine, methylester (TMRM) as the probe. A cell density of 5×10^3^ HCT 116 cells per well (96 well plate) was used in the study. Briefly, the cells were incubated for 24 h with plain-E, PIC-raw, and PIC-E with reference to 0.1 µM PIC. The cells were kept in the working solution of the probe after removing the medium in cells and were then incubated in the dark. Finally, the probe solution was replaced with the live-cell imaging buffer and analyzed using FACSCalibur, BD Bioscience flow cytometer [17,18,19]. 

#### 2.3.7. Cleaved Caspase-3 Content 

A quantitative assay for caspase-3 was performed using a commercially available kit (ABCAM, Cambridge, UK). After incubation of the samples with the HCT 116 cells (5×10^3^ cells per well), the cells were washed and lysed. Finally, cell lysate was treated as recommended by the kit manufacturer and the absorbance was assessed at 405 nm to quantify the caspase-3 concentration expressed as pg per mg protein which was determined using the BCA Protein Assay Kit (Sigma–Aldrich, St. Louis, MO, USA) [20,21].

#### 2.3.8. Real-Time Polymerase Chain Reaction

Qiagen’s RNeasy Mini Kit (Qiagen, UK) was used for RNA extraction from liver tissues. After RNA normalization of all the tubes to 2 μg, reverse transcription with SuperScript III cDNA Synthesis System (Invitrogen, UK) to complementary DNA (cDNA) was carried out in a 20 μL reaction mix. Gene Runner software was used for primer design. Primers had sequences of different exons with spanning and flanking of introns to exclude the amplification of contaminating genomic DNA (gDNA). Table 3 gives the primer sequences for β-actin, *Bax*, and *Bcl-2*. Relative expression patterns of assessed genes were carried out with 1 μL synthesized cDNA (10 ng/μL) as the template in 5 μL Power Up SYBR Green PCR Master Mix and 0.75 μL of each primer using a 7500 Fast real-time PCR system (Applied Biosystems). β-Actin was selected as the housekeeping gene. Results were validated using the relative quantification (ΔΔCt) method. The genes were measured in triplicates and the mean for these runs was normalized with the mean of β-actin (Table 3).

#### 2.3.9. Assessment of Nitric Oxide (NO) Production 

A Griess reagent-based colorimetric test was used for determining sodium nitrite (NaNO2^−^) accumulation in the culture medium as an indicator of nitric oxide production. Fifty microliter samples were collected after 24 h treatment of HCT 116 cells with plain-E, PIC-raw or PIC-E at concentrations equivalent to 0.1 µM of PIC. Griess reagent (Sigma–Aldrich, Saint Louis, MO, USA) and culture medium were mixed in equal volumes. Absorbance was recorded by a microplate reader at 550 nm after a 10 min incubation at room temperature. A sodium nitrite standard curve was employed for the determination of nitrite concentrations in a range of 26–100 mmol/L NaNO_2_. 

#### 2.3.10. Reactive Oxygen Species (ROS) Determination 

For the determination of ROS, 96 well plates with a cell density of 5×10^3^ HCT 116 cells/well were prepared and incubated with plain-E, PIC-raw or PIC-E at concentrations equivalent to 0.1 µM of PIC for 24 h. Samples were studied in comparison with untreated cells as the control. Cell staining was carried out using 10 μM 2,7-dichlorofluorescein diacetate (DCFDA) for 45 min using a commercially available kit (ABCAM, Cambridge, UK). This was followed by washing with PBS before the determination of fluorescence using a spectrofluorometer with excitation/emission at 485 nm/535 nm [22]. 

#### 2.3.11. Statistical Analysis 

Data are presented as mean ± SD. The IBM SPSS^®^ statistics software, version 25 (SPSS Inc., Chicago, IL, USA), was used for statistical analysis. Means were compared using one-way analysis of variance (ANOVA) followed by Tukey as a post-hoc test. Values of *p* < 0.05 were considered significant.

## 3. Results

### 3.1. Statistical Analysis for Model Selection

**Figure 1:**
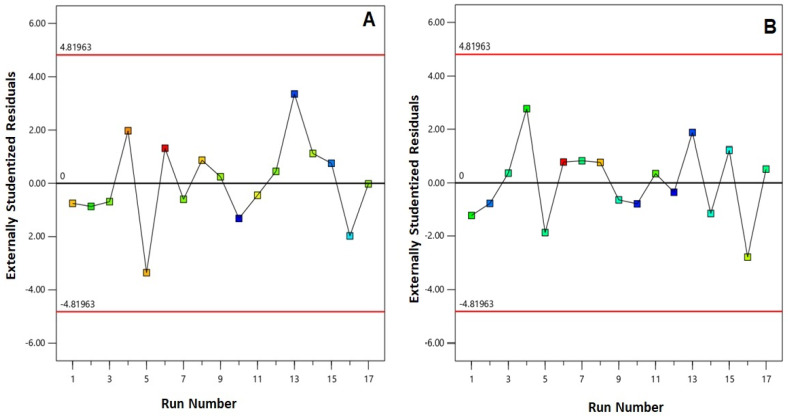
Externally studentized residual plot versus run for (**A**) particle size and (**B**) entrapment efficiency% of PIC-E.

In this study, a three-factor, three-level Box–Behnken design was employed for formulation and of PIC-E and their optimization with minimized particle size and maximized drug entrapment. The combinations at the center and the mid points of the edges of the design space represent the combination of variables for the proposed experimental runs. The best fitting model was the quadratic model for both responses based on its highest determination coefficient *R*^2^. The value of predicted *R*^2^ reasonably agrees with the adjusted *R*^2^ indicating the validity of the model. An adequate precision of greater than 4 indicates a low signal-to-noise ratio, thus highlighting the suitability of the selected model to navigate the design space (Table 4). Figure 1 plots the externally studentized residuals versus run order for both entrapment efficiency and particle size. The random scatter of the plot indicates the absence of any lurking variables that may have affected the response. This confirms the model validity and the effectiveness of the randomization process. 

#### 3.1.1. Variables’ Influence on Particle Size

The prepared PIC-E exhibited nano-vesicular sizes ranging from 99.31 ± 2.12 to 212.32 ± 7.32 nm. The relatively small standard deviation indicated the homogeneity of distribution. In addition, the existence of the size in the nano-range could have a crucial effect on enhancing the drug targeting to the cancer cells. Analysis of variance ensured the significance of the quadratic model as evidenced by its F-value of 131.06 (*p* < 0.0001). The non-significant lack of fit (*p* = 0.1069) confirmed fitting of the response to the proposed model. The polynomial equation conforming to the sequential model was generated in terms of coded factor as such:

Y = +171.88 + 10.60 X_1_ + 43.48 X_2_ − 3.17 X_3_ − 2.40 X_1_X_2_ + 1.01 X_1_X_3_ + 1.20 X_2_X_3_ + 5.30 X_1_^2^ − 18.99 X_2_^2^ − 1.89 X_3_^2^

#### 3.1.2. Variables’ Influence on Particle Size and PIC-E

**Figure 2:**
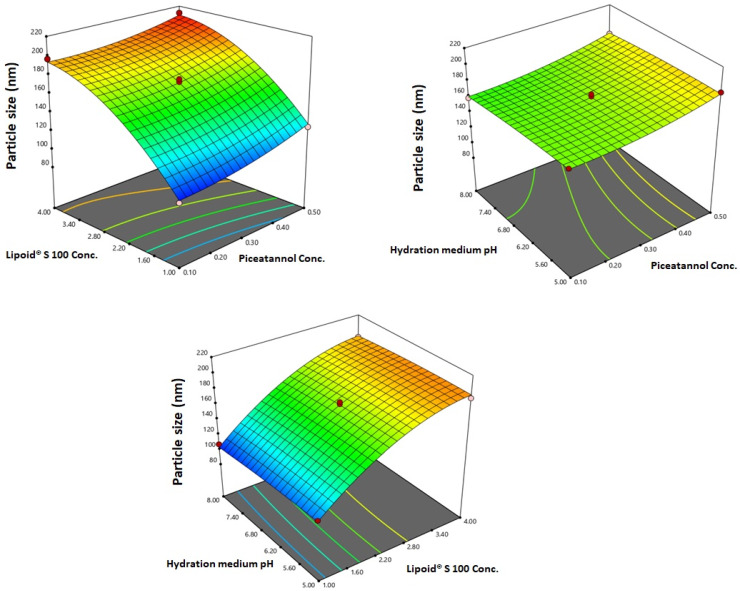
3D response surface plots for the effect of PIC concentration, Lipoid^®^ S 100 concentration, and the pH of the hydration medium on the particle size of PIC-E.

The prepared PIC-E exhibited nano-vesicular sizes ranging from 99.31 ± 2.12 to 212.32 ± 7.32 nm. Figure 2 illustrates the 3D response surface plots for the effect of the investigated variables on the particle size. 

The relatively small standard deviation indicated homogeneity of distribution. In addition, the existence of the size in the nano-range could have a crucial effect on enhancing the drug targeting to the cancer cells. Analysis of variance (ANOVA) ensured the significance of the quadratic model as evidenced by its F-value of 131.06 (*p* < 0.0001). The non-significant lack of fit (*p* = 0.1069) confirmed the fitting of the response to the proposed model. The polynomial equation conforming to the sequential model was generated in terms of coded factor as such:

Y = +171.88 + 10.60 X_1_ + 43.48 X_2_ − 3.17 X_3_ − 2.40 X_1_X_2_ + 1.01 X_1_X_3_ + 1.20 X_2_X_3_ + 5.30 X_1_^2^ − 18.99 X_2_^2^ − 1.89 X_3_^2^

#### 3.1.3. Variables’ Influence on Particle Size

The entrapment efficiency of the prepared PIC-E ranged from 81.21% ± 2.17% to 96.51% ± 1.99%. The significance of the quadratic model was confirmed by the F-value of 61.60 computed by ANOVA test (*p* = 0.0007). The non-significant lack of fit (*p* = 0.1821) confirmed fitting of the response to the proposed model. The polynomial equation conforming to the sequential model was generated in terms of coded factor as such:

Y = +86.54 + 4.67 X_1_ + 2.49 X_2_ − 0.35 X_3_ − 0.60 X_1_X_2_ − 1.32 X_1_X_3_ + 1.28 X_2_X_3_ + 2.13 X_1_^2^ + 0.98 X_2_^2^ − 1.70 X_3_^2^

### 3.2. Optimization of PIC-E

**Figure 3:**
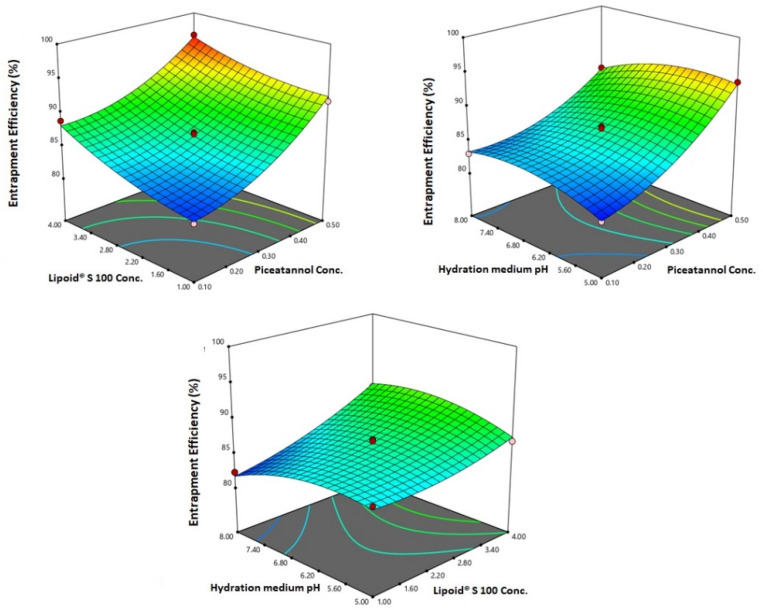
3D response surface plots for the effect of PIC concentration, Lipoid^®^ S 100 concentration, and pH of hydration medium on entrapment efficiency % of PIC-E.

The optimized levels of the variables were predicted using numerical optimization with a desirability of 0.775. Preparation and evaluation of the optimized formulation was carried out. A low residual error between the predicted and actual responses confirmed the success of the optimization technique. The optimized formulation’s variables levels, predicted, and observed responses are depicted in Table 5. Figure 3 illustrates the 3D response surface plots for the effect of the investigated variables on the entrapment efficiency.

### 3.3. Characterization and Evaluation of Optimized Formulation

#### 3.3.1. Transmission Electron Microscopy

**Figure 4:**
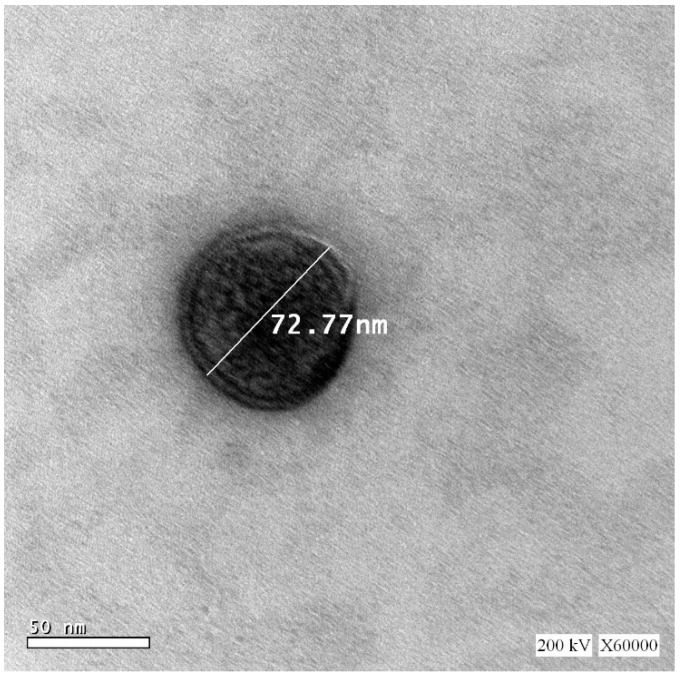
TEM image of PIC-E (X 60,000).

The TEM image of PIC-E is represented in Figure 4. The size of emulsomes was consistent with that obtained from the DLS technique. The emulsomes appeared almost spherical with no aggregation. The ridges that appeared on the emulsomes might be due to the shrinkage of the lipid surface layer during the drying process in the sample preparation. The observed results were similar to those reported emulsomes [21,22].

#### 3.3.2. Drug Release (In Vitro)

**Figure 5:**
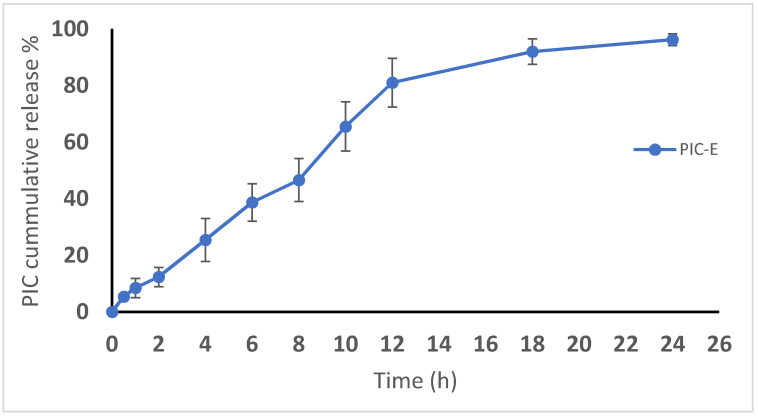
In vitro release profile of PIC-E in phosphate-buffered saline (pH 7.4, 0.1 M) containing Tween 80 (0.1%) at 37 ± 0.5 °C.

The in vitro dissolution of PIC from the emulsomes was studied. Figure 5 shows the drug release profile. A satisfactory gradual release profile was observed for the prepared emulsomes. Approximately 50% of drug release was observed at 8 h. The drug release was almost complete by 24 h.

#### 3.3.3. Determination of IC_50_ Values 

The IC_50_ values were determined by MTT assay using HCT 116 cells. The results indicated a significant reduction of the IC_50_ value for the PIC-E when compared to PIC-raw. The IC_50_ value for the PIC-E was 7.02 ± 0.23 µM, while it was 18.94 ± 1.91 µM for the PIC-raw, and plain-E showed an IC_50_ value of 118.3 ± 5.4 µM (Table 6). Further, the optimized formula was examined in an addental colon cancer cells HCT 29. Plain-E, PIC raw, and PIC E showed IC_50_ of values 131.0 ± 2.6, 18.4 ± 1.7, 6.3 ± 0.2. All preparations exhibited IC_50_ vales greater than 30 µM against EA.hy926 non-cancerous endothelia cells. 

#### 3.3.4. Cell Cycle Analysis

**Figure 6:**
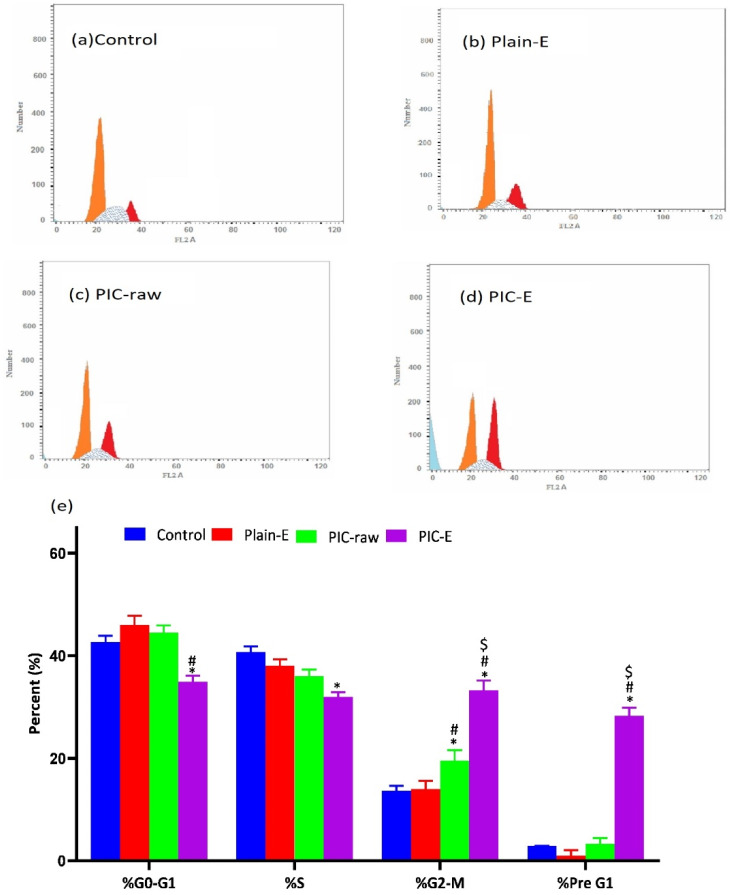
Impact of PIC-E on cell cycle phases of HCT 116 cells. (**a**) Control, (**b**) Plain-E, (**c**) PIC-raw, (**d**) PIC-E, and (**e**) bar diagram of the different cycle phases. Cells were incubated for 24 h with plain-E, PIC-raw (0.1 µM) or PIC-loaded E (equivalent to 0.1 µM PIC). * Significantly different from control at *p* < 0.05. ^#^ Significantly different from plain-E at *p* < 0.05. ^$^ Significantly different from PIC-raw at *p* < 0.05.

The optimized formula caused accumulation of the cells in the G2-M phase as well as significant increases in the cell fractions detected in the sub-G1 phase (Figure 6). 

#### 3.3.5. Annexin V Staining

**Figure 7:**
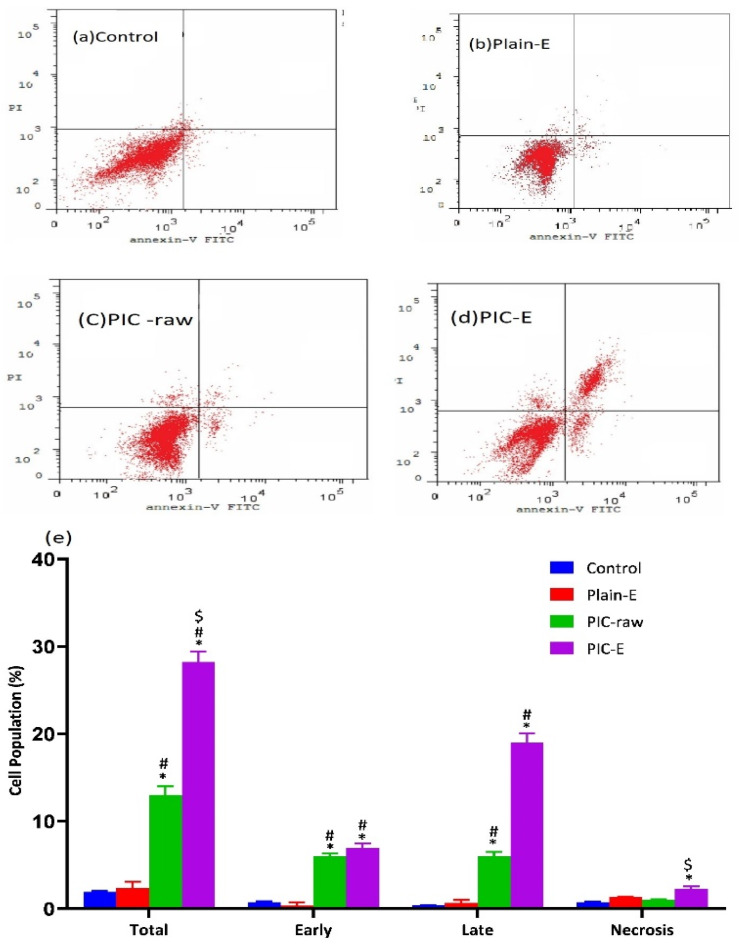
Effect of PIC-E on apoptotic and necrotic death in HCT 116 cells and impact of PIC-E on cell cycle phases of HCT 116 cells: (**a**) Control, (**b**) Plain-E, (**c**) PIC-raw, (**d**) PIC-E, and (**e**) bar diagram of different types of cell death. Cells were incubated for 24 h with plain-E, PIC-raw (0.1 µM), or PIC-E (equivalent to 0.1 µM PIC) * Significantly different from control at *p* < 0.05. ^#^ Significantly different from Plain-E at *p* < 0.05.

The data in Figure 7 show the influence of the optimized PIC formula on the different types of cell death via apoptosis as well as necrosis. Piceatannol-emulsomes significantly enhanced early and late apoptosis, necrosis, and total cell death.

#### 3.3.6. Mitochondrial Membrane Potential

**Figure 8:**
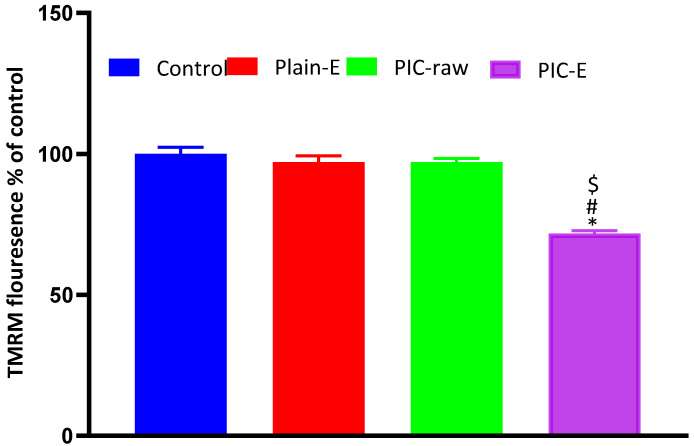
Effect of PIC-E on MMP in HCT 116 cells. Cells were incubated for 24 h with plain-E, PIC-raw (0.1 µM), or PIC-E (equivalent to 0.1 µM PIC) * Significantly different from control at *p* < 0.05. *^#^* Significantly different from plain-E at *p* < 0.05. ^$^ Significantly different from PIC-raw at *p* < 0.05.

Accession of TMRM into mitochondria is an indicator of MMP. Thus, retaining high fluorescence is an indicator of healthy mitochondria. Cells undergoing apoptosis have relatively depolarized mitochondrial membranes. Therefore, decreased fluorescent staining of TMRM is an indirect indicator of mitochondrial membrane disturbing; our data indicate that PIC-raw did not have any significant effect on MMP as compared to the control values. Only PIC-E significantly reduced MMP (Figure 8). 

#### 3.3.7. Cleaved Caspase-3 Content 

**Figure 9:**
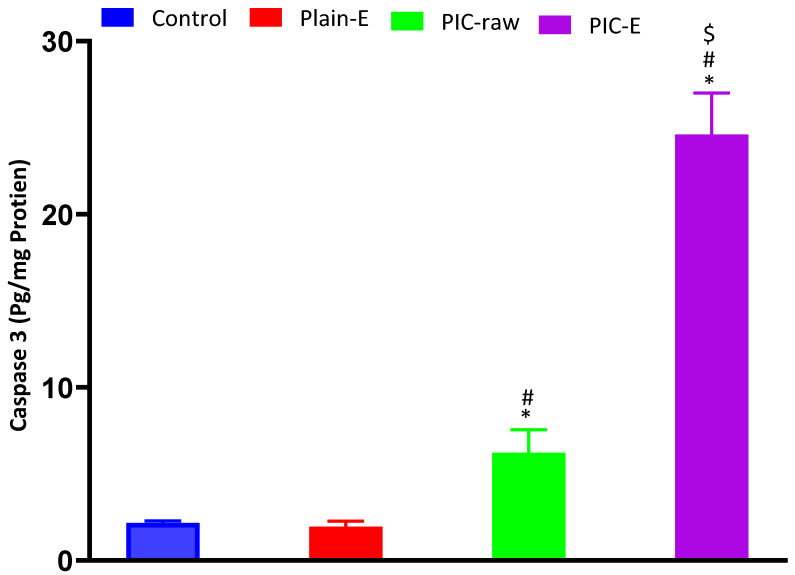
Effect of PIC-E on cleaved caspase-3 content in HCT 116 cells. Cells were incubated for 24 h with plain-E, PIC-raw (0.1 µM), or PIC- E (equivalent to 0.1 µM PIC) * Significantly different from control at *p* < 0.05. # Significantly different from plain-E at *p* < 0.05. $ Significantly different from PIC-raw at *p* < 0.05.

Figure 9 indicates that PIC and the optimized PIC-E formula significantly enhanced caspase-3 content in HCT 116 cells. Cells treated with the optimized formula exhibited significantly higher content of cleaved caspase-3 as compared to those challenged with PIC-raw.

#### 3.3.8. mRNA Expression of *Bax* and *Bcl-2*

**Figure 10:**
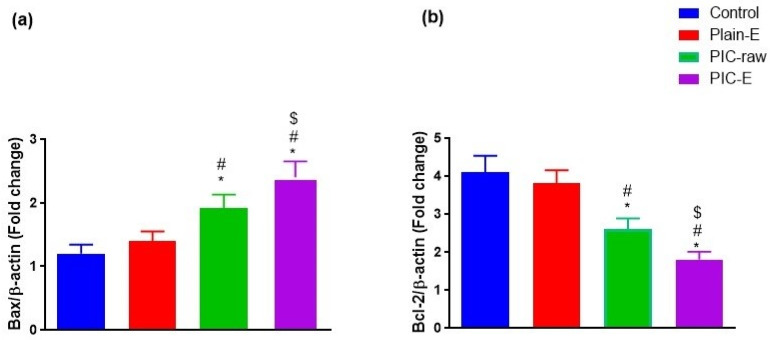
Effect of PIC-E on *Bax* and *Bcl-2* mRNA expression content in HCT 116 cells; cells were incubated for 24 h with plain-E, PIC-raw (0.1 µM), or PIC- E (equivalent to 0.1 µM PIC) * Significantly different from control at *p* < 0.05. ^#^ Significantly different from plain-E at *p* < 0.05. ^$^ Significantly different from PIC-raw at *p* < 0.05.

Assessing mRNA expression of *Bax, Bcl-2*, and their ratio that confirms the proapoptotic activity was confirmed by the data in Figure 10. The PIC-E significantly upregulated *Bax* and downregulated *Bcl-2* expression. 

#### 3.3.9. Nitric Oxide Determination

**Figure 11:**
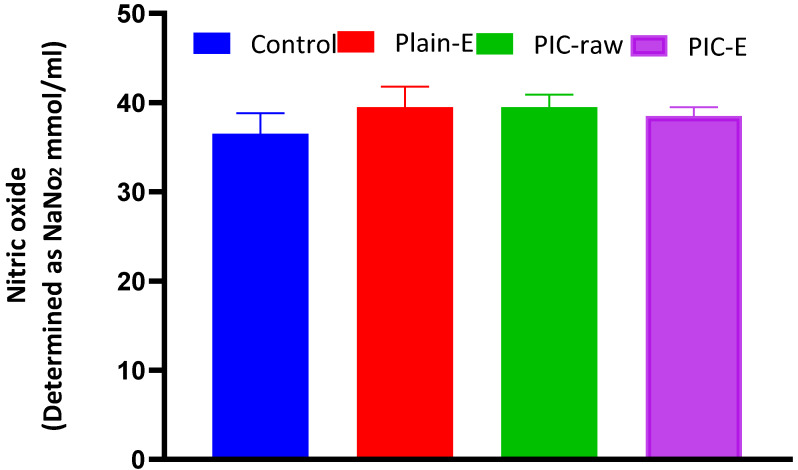
Effect of PIC-E on nitric oxide production in HCT 116 cells. Cells were incubated for 24 h with plain-E, PIC-raw (0.1 µM), or PIC- E (equivalent to 0.1 µM PIC).

In this study, no significant effect on nitric oxide production was observed with PIC or PIC-E (Figure 11).

#### 3.3.10. ROS Determination

**Figure 12:**
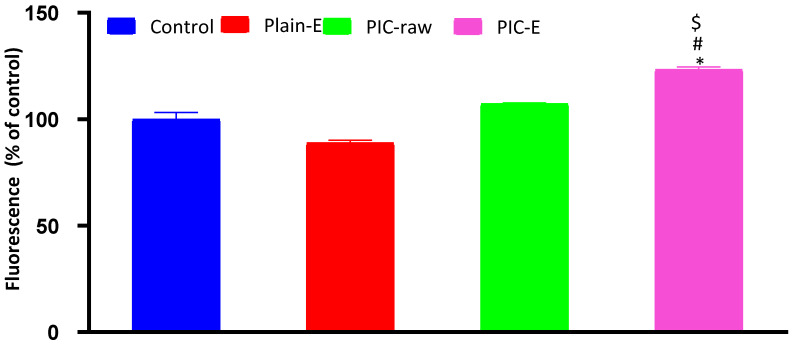
Effect of PIC-E on ROS generation in HCT 116 cells. Cells were incubated for 24 h with plain-E, PIC-raw (0.1 µM), or PIC-E (equivalent to 0.1 µM PIC) * Significantly different from control at *p* < 0.05. ^#^ Significantly different from Plain-E at *p* < 0.05. ^$^ Significantly different from PIC-raw at *p* < 0.05.

Conversion of DA to fluorescent DCF was used for the determination of ROS in the current study. Only the optimized PIC-E significantly increased ROS production when compared to the control, plain-E, and PIC-raw groups (Figure 12). 

## 4. Discussion

The ANOVA showed a positive and significant effect of the linear terms X1 (PIC concentration) and X2 (lipoid concentration) on the particle size at *p* < 0.05 as evidenced by the positive coefficients of these terms in the generated equation. In addition, the quadratic term X12 and X22 corresponding to the same variables were significant at the same level. The increase in particle size with higher drug concentrations might be caused by the observed increased drug entrapment, while the increase in size with higher phospholipid concentration could be ascribed to the multiple bilayer formations. This observation was in accordance with previous studies. Vyas et al. [23] reported an increase in the particle size of liver-targeted emulsomes of zidovudine upon increasing molar ratio of soya phosphatidylcholine. They also reported a significant increase in oxycarbamazepine emulsomes on increasing phosphatidylcholine to triglycerides ratio [24,25,26,27]. The ANOVA showed that the drug entrapment within the emulsomes increases significantly with increasing PIC and lipoid concentrations at *p* < 0.05 as evidenced by the positive coefficients of the linear terms linear terms X1 and X2 [28]. The quadratic term corresponding to the three variables as well as the interaction terms X1X3 and X2X3, corresponding to the interaction between the pH of the hydration medium and either the drug or lipoid concentrations, were also significant at the same significance level. Increased entrapment with higher PIC and lipoid concentrations could be credited to the lipophilic properties of the drug. Thus, a higher lipid concentration could result in enhanced drug dissolution in the lipids and, consequently, improved drug entrapment [29]. The release could be considered well enhanced considering the poor solubility of the PIC. The in vitro release of PIC encapsulated in albumin nanoparticles, too, was found to be very low compared to our results [9]. Albumin nanoparticles could release only approximately 20%-40% of PIC loaded for various formulations. The observed higher drug release observed in our study could be explained by the solubilizing power of emulsomes towards the hydrophobic drug by virtue of the lipophilic nature of its components. The emulsomes ability to enhance the solubility of other poorly water-soluble drugs has been reported [14]. The PIC-E exhibited a one-fold decrease in the IC_50_ value as compared to PIC-raw. This is similar to a previous study showing the cytotoxic activity of PIC in HCT 116 cells [30]. These cytostatic activities of PIC-E were confirmed in HTT 29 colon cancer cells. Fortunately, the optimized formula showed relatively week cytotoxic activity against EA.hy926 non-cancerous endothelial cells. It could be concluded that the cytotoxicity of PIC was greatly enhanced by its formulation of emulsomes. High cellular permeability could provide an explanation for the enchanted cytotoxicity. The suggested high permeability could be understood on the basis of the lipophilic nature of the delivery system along with the nano-size range [31,32]. Assessing the impact of PIC-E on cell cycle phases revealed the accumulation of cells in the G2-M phase and increased cell fraction in the sub-G1 phase; these data are supported from a previous report [33] on PIC that highlighted the ability of PIC to cause SK-Mel-28 melanoma cell arrest in the G2-M phase, an effect attributed to cyclins A, E, and B1 downregulation. Also, PIC significantly increased the percentage of apoptotic cells in the pre-G phase [34]. Nevertheless, reported studies have also indicated that PIC blocks cells in the G0–G1 phase [35]. This may be explained by the relatively low concentrations used in the present study. The observed increased fraction of HCT 116 cells in the sub-G1 phase highlights enhanced potent pro-apoptotic activity. This is in line with the same previous studies suggesting induction of apoptosis as a mechanism of anti-proliferative properties of PIC [36]. In this regard, it is noteworthy to report the superiority of PIC-E as compared to pure PIC. This confirms a role for emulsomes in boosting PIC pro-apoptotic activities. In regard to annexin V, these data gain support from previous reports showing enhanced melanoma cell staining with annexin V after challenge with PIC. In addition, PIC-E showed significant increase in the proportion of HCT 116 cells with positive annexin staining. This is in harmony with studies showing the ability of emulsomes to enhance annexin V-staining of HCT 116 cells when loaded with curcumin and piperine [37]. These observations might be due to the lipophilic nature of the formulation, which offers improved delivery of the anti-proliferative agents [38]. In the current study, only PIC formulated in emulsomes significantly disturbed MMP as indicated by the mitochondrial entrapment of the fluorescent dye TMRM. This is due to the cationic and lipophilic nature of the dye [39]; during apoptosis, mitochondrial permeability increased and the mitochondria become depolarized [40]. In other words, mitochondria of apoptotic cell retain less fluorescence. Several previous studies indicated the ability of PIC to disrupt MMP as a step eventually leading to apoptosis [41,42]. Depending on the structure similarity to resveratrol, these data give support to the oxidative stress observed. Assessing MMP indicated that PIC-raw did not show significant effects in the current study. This may be due to the much lower concentrations of PIC used in herein. Use of emulsomes as carriers signified the PIC effect; the matter that points the efficiency of emulsomes as carriers of cancer cell death-inducing agents. Also, the structurally related compound resveratrol has been reported to stimulate production of nitric oxide [43]. 

In the present study, PIC-E significantly enhanced caspase-3 content. This is in harmony with previous reports indicating the ability of PIC to enhance cellular caspase-3 [41,42]. In addition, the enhanced effects of emulsomes on cleaved caspase-3 content in HCT 116 cells has been previously demonstrated [37]. Elevation of cleaved caspase-3 content is the last cytosolic event preceding apoptosis. Thus, it can be deduced that formulating PIC in a nanostructured system greatly enhances caspase-3 content. Indeed, boosting of cleaved caspase-3 activity by nanostructured systems of anti-tumor agents has been well reported [44,45,46]. The proapoptotic activities of PIC-E was confirmed by assessing *Bax* and *Bcl-2* mRNA expression. The optimized formula exhibited significant upregulation of *Bax* and downregulation of *Bcl-2* mRNA expression. These results support the observed enhancement of caspase-3 content. Further, PIC-E significantly increased oxidative stress in colon cancer cells. In general, phenolic compounds can acquire a pro-oxidant behavior under certain conditions that depend on pH and the availability of redox-active metals [47] and form an aroxyl radical that can lead to the generation of superoxide anions [44]. Since transition metals are more represented in cancer than in normal cells, polyphenols can be engaged in Fenton and Fenton-like reactions and generate hydroxyl radicals [34]. In this regard, PIC has been shown to induce oxidative stress in HeLa and A375SM cancer cells [44,46]. 

Nitric oxide concentrations were comparable with control values. Nitric oxide plays an essential role in the proliferation and metastasis of cancer cells [30]. It has been established that PIC promotes endothelial nitric oxide synthase activity [48]. On the other hand, several reports indicated that the anti-inflammatory properties of PIC result from the inhibition of nitric oxide synthesis [30]. In addition, it has been shown that PIC inhibited nitric oxide production and iNOS activity in experimental models [49,50]. These data are integral to our observation on cell cycle phases, annexin V staining, and caspase-3 concentration. Thus, the impact of PIC on nitric oxide seems to depend on the context of cellular microenvironment. 

## 5. Conclusions

In the present study, a successful application of the Box–Behnken design was used in the formulation and optimization of PIC-E. Prepared emulsomes exhibited nano-size and high drug entrapment exceeding 80%. The optimized emulsomes formulation with minimized particle size and maximized drug entrapment exhibited a spherical shape with gradual and complete in vitro release. PIC release (in vitro) indicated the ability of PIC-E to improve the dissolution of the poorly soluble drug. The PIC-E exhibited superior cell death-inducing activities against HCT 116 cells as compared to PIC-raw. This is mediated, at least partially, by enhanced pro-apoptotic activity, disruption of MMP, and stimulation of ROS generation. 

## Figures and Tables

**Table 1:** Independent variables and responses used in the Box–Behnken design for the formulation and optimization Piceatannol-emulsomes (PIC-E).

**Independent Variables**	**Levels**		
	**(−1)**	**(0)**	**(+1)**
X_1_: PIC concentration (%w/w)	0.10	0.30	0.50
X_2_: Lipoid^®^ S 100 concentration (%w/w)	1.00	2.50	4.00
X_3_: pH of hydration medium	5.00	6.50	8.00
**Responses**	**Desirability constraints**		
Y1: Particle size (nm)	Minimize		
Y2: Entrapment efficiency (%)	Maximize		

**Table 2:** Experimental runs, variables levels, and responses observed of PIC-E prepared based on a Box–Behnken design.

Run #	Independent Variables			Particle Size (nm) ± SD	Entrapment Efficiency (%) ± SD
	PIC Concentration (%w/w)	Lipoid^®^ S 100 Concentration (%w/w)	Hydration Medium pH		
F1	0.30	4.00	8.00	191.10 ± 3.45	88.81 ± 1.45
F2	0.10	2.50	8.00	158.81 ± 2.98	82.98 ± 1.99
F3	0.30	2.50	6.50	169.43 ± 3.99	86.81 ± 2.31
F4	0.10	4.00	6.50	196.75 ± 4.53	88.83 ± 2.16
F5	0.30	4.00	5.00	192.32 ± 6.54	86.82 ± 1.56
F6	0.50	4.00	6.50	212.32 ± 7.32	96.51 ± 1.99
F7	0.30	2.50	6.50	169.72 ± 3.51	87.10 ± 2.54
F8	0.50	2.50	5.00	189.76 ± 5.14	93.61 ± 2.32
F9	0.30	2.50	6.50	172.80 ± 5.89	86.19 ± 2.27
F10	0.10	1.00	6.50	99.31 ± 2.12	81.60 ± 1.49
F11	0.50	2.50	8.00	182.81 ± 8.32	90.12 ± 1.89
F12	0.10	2.50	5.00	169.83 ± 4.46	81.21 ± 2.17
F13	0.30	1.00	8.00	107.34 ± 2.79	82.30 ± 2.39
F14	0.30	2.50	6.50	175.71 ± 7.45	85.82 ± 1.39
F15	0.30	1.00	5.00	113.42 ± 3.11	85.41 ± 1.83
F16	0.50	1.00	6.50	124.50 ± 3.62	91.70 ± 1.92
F17	0.30	2.50	6.50	171.81 ± 4.19	86.91 ± 2.71

**Table 3:** Nucleotide sequences of the primers used for the analysis of mRNA expression by qRT-PCR.

Gene	Primer Sequence from 5′–3′
*β-actin*	F: TCCGTCGCCGGTCCACACCC R: TCACCAACTGGGACGATATG
*Bax*	F: CCTGAGCTGACCTTGGAGCA R: GGTGGTTGCCCTTTTCTACT
*Bcl-2*	F: TGATAACCGGGAGATCGTGA R: AAAGCACATCCAATAAAAAGC

**Table 4:** Quadratic model statistics of PIC-E responses.

Responses	Sequential *p*-Value	Lack of Fit *p*-Value	*R* ^2^	Adjusted *R*^2^	Predicted *R*^2^	Adequate Precision	PRESS *	Significant Terms
Y1: Vesicle size (nm)	0.0001	0.1069	0.9941	0.9865	0.9269	36.3396	1305.69	X1, X2, X12, X22
Y2: Entrapment Efficiency (%)	0.0007	0.1821	0.9864	0.9689	0.8476	26.4061	42.26	X1, X2, X1X3, X2X3, X12, X22, X32

* PRESS, predicted residual error sum of squares.

**Table 5:** Optimized variables’ levels of optimized PIC-E and its predicted and observed responses.

**Variables**	**X1: PIC Concentration (w/w)**	**X2: Lipoid^®^ S 100 Concentration (w/w)**	**X3: Hydration Medium pH**
Optimum values	0.50	1.00	5.20
	Predicted value	Observed value	Residual
Vesicle size (nm)	129.21	125.45	−3.76
Entrapment efficiency (%)	93.71	93.14	−0.57

**Table 6:** Cytotoxicity of PIC-E in HCT 116, HCT 29, and EA.hy926 cells.

Samples-	IC_50_ Value (µM)		
	**HCT 116**	**HCT 29**	**EA.hy926**
Plain- E	118.3 ± 5.4	131.0 ± 2.6	159.8 ± 3.6
PIC-raw	18.9 ± 1.9 ^*^	18.4 ± 1.7 ^*^	50.7 ± 4.1 ^*^
PIC-E	7.02 ± 0.2 ^* #^	6.3 ± 0.2 ^* #^	38.6 ± 3.2 ^* #^

Cells were incubated with plain-E, PIC-raw or PIC-E for 48 h, and IC_50_ values were determined using an MTT assay. Data are presented as ±SD. * Significantly different from plain-E at *p* < 0.05. ^#^ Significantly different from PIC-raw at *p* < 0.05.
